# Editorial: Neural differentiation and neurorestorative effects of mesenchymal stem cells in brain disorders

**DOI:** 10.3389/fncel.2026.1954548

**Published:** 2026-08-18

**Authors:** Ivana R. Romano, Dirk M. Hermann, Giuliana Mannino, Debora Lo Furno

**Affiliations:** 1 Department of Biomedical and Biotechnological Sciences (BIOMETEC), University of Catania, Catania, Italy; 2 Department of Neurology, University Hospital Essen, University of Duisburg-Essen, Essen, Germany; 3 Department of Medicine and Surgery, University of Enna “Kore”, Enna, Italy

**Keywords:** extracellular vesicles (EVs), mesenchymal stromal/stem cells (MSCs), neurological disorders, neuroprotection, regenerative medicine

Mesenchymal stromal/stem cells (MSCs) have emerged as one of the most promising cell population in regenerative medicine due to their multilineage differentiation potential, low immunogenicity and, above all, their broad immunomodulatory and trophic activities. Although MSCs have traditionally been recognized for their ability to differentiate into mesodermal tissues, increasing evidence has demonstrated their neural plasticity and, more importantly, their ability to promote neuroprotection and tissue repair through paracrine mechanisms (Lee et al., 2025; Uccelli et al., 2011; Phinney and Pittenger, 2017). This evolving concept has substantially influenced the development of regenerative strategies to overcome the limited regenerative capacity of the central nervous system.

The aim of this Research Topic is to improve our understanding on the biological mechanisms underlying the therapeutic potential of MSCs in neurological diseases, with particular emphasis on two key aspects: their capacity for neural differentiation and their neuroprotective/neurorestorative properties.

This Research Topic include four reviews, two original articles and a case report. The papers address the molecular mechanisms that regulate neural commitment, the comparative analyses of MSCs derived from different tissue sources, the role of extracellular vesicles mediating therapeutic effects, the innovative strategies to enhance MSC efficacy.

## Reviews

The review articles provide a comprehensive overview of the current landscape of MSC-based therapies for neurological disorders, covering both clinical evidence and biological mechanisms underlying their therapeutic potential.

Neurological disorders represent an increasing proportion of global diseases due to extended population aging and the limited availability of efficacious therapies (Huang et al., 2023; GBD 2023 Road Injuries Collaborators, 2026). Despite significant advances in symptomatic management, neurological disorders still represent a long-term disability, underscoring the urgent need for innovative approaches. Lepski and Arévalo present an evidence-mapped review of clinical studies. Their analysis highlights the encouraging safety profile of MSC administration in a broad spectrum of diseases, including stroke, traumatic injuries, neurodegenerative disorders, and multiple sclerosis. The authors also underline that the therapeutic efficacy remains difficult to establish because of considerable heterogeneity in study design, cell sources, manufacturing procedures and administration routes.

According to the increasing evidence indicating that MSC therapeutic efficacy is often mediated through paracrine mechanisms (Uccelli et al., 2011; Phinney and Pittenger, 2017), the review by Nakazaki et al., focuses on extracellular vesicles (EVs). Rather than considering MSCs solely as replacement cells, the authors report that EVs are main mediators of MSC-related effects. By transferring microRNAs, proteins, lipids, and other regulatory molecules, EVs modulate neuroinflammation, attenuate apoptosis, promote angiogenesis and neurogenesis, and enhance synaptic plasticity in several experimental models of neurological diseases (Colombo et al., 2014; Cunningham et al., 2018; Welsh et al., 2024).

Auditory neuropathy is a debilitating form of sensorineural hearing loss characterized by the degeneration or dysfunction of spiral ganglion neurons (SGNs). Based on existing literature (You et al., 2026; Gunewardene et al., 2014), Aasar et al. compare MSCs with induced pluripotent stem cells (iPSCs) for SGN regeneration in auditory neuropathy. While MSCs are recognized for their favorable safety profile, iPSCs exhibit superior differentiation potential but remain associated with unresolved issues regarding maturation, tumorigenicity, and clinical safety. Their comparative analysis illustrates how different stem cell populations may offer complementary therapeutic opportunities depending on the biological and clinical context. This perspective aligns with current efforts to optimize stem cell selection for neurological regeneration.

Alzheimer's disease is the most common cause of dementia and currently available therapies offer only limited symptomatic benefit. Preclinical evidence indicates that human umbilical cord MSCs (hUCMSCs) can reduce amyloid pathology, enhance neuroplasticity, and improve cognitive performance through multiple complementary mechanisms (Frisoni et al., 2025; Heneka et al., 2024). Si et al. provide a systematic review evaluating the efficacy of hUCMSCs in mouse models of the disease. Their meta-analysis demonstrates significant improvements in cognitive performance together with reductions in amyloid-β deposition, tau phosphorylation, and neuroinflammation. Although the authors acknowledge the methodological heterogeneity among the included studies, the findings further support that MSCs exert multifaceted neuroprotective effects.

Collectively, these reviews encompass the major objectives of this Research Topic, integrating current clinical evidence, mechanistic insights, comparative stem cell biology, and preclinical therapeutic outcomes, also identifying the challenges that remain for successful clinical translation.

## Research articles

The original research articles in this Research Topic investigate novel strategies aimed at enhancing the therapeutic efficacy of MSC-based approaches, also clarifying the molecular mechanisms underlying their neuroprotective properties.

Ischemic stroke is one of the main causes of death and long-term disability, and current reperfusion therapies are limited by narrow therapeutic windows and their inability to prevent secondary injury (He et al., 2024). Huang et al. demonstrate that conditioned medium derived from adipose MSCs significantly attenuates ischemia-induced neuronal injury. The authors claim that the activation of the JAK1/STAT3 signaling pathway is a critical mediator of the observed neuroprotective effects, providing additional evidence that the therapeutic potential of MSCs largely depends on their secretome.

As neural regeneration is strongly impaired by the limited spontaneous central nervous system repair (Andrzejewska et al., 2021; Wang et al., 2025), efficacious strategies need to be explored. Recently, three-dimensional MSC spheroids and photobiomodulation (PBM) have reported capable of enhancing stem cell survival, secretory activity, and regenerative efficacy (Petrenko et al., 2017; Hamblin, 2018). In this field, Chang et al. show that PBM preconditioning enhances the viability of human MSC spheroids, increases the secretion of neurotrophic factors, and also potentiates their neuroprotective capacity in experimental models. Their work illustrates how bioengineering approaches can improve the regenerative performance of MSCs before transplantation, representing an attractive strategy for optimizing future cell-based therapies.

## Case report

Hypoxic–ischemic brain injury is one of the most devastating neurological conditions. Despite the feasibility and short-term safety of MSC administration in neurological disorders, robust evidence of efficacy remains limited and requires further validation in rigorous clinical trials (Wada et al., 2025; Harris et al., 2024; Bydon et al., 2024). The case report by Lepski et al. provides an example of early clinical translation by describing the compassionate use of autologous bone marrow MSCs in a patient with chronic anoxic encephalopathy. The authors report that the treatment was feasible and well-tolerated, provided neurological improvements during follow-up, without serious treatment-related adverse events. Although the limitations inherent to a single-patient observation preclude definitive conclusions, this report supports the growing evidence that MSC administration is clinically practicable and further emphasizes the need for rigorously designed controlled clinical trials.

Overall, the original contributions successfully address the aims of this Research Topic, by expanding our understanding on MSC-mediated neuroprotection, also identifying novel strategies to enhance therapeutic efficacy, and promoting the translation of experimental findings into clinically relevant approaches.

We sincerely thank all the authors, reviewers, and editors whose valuable contributions made this Research Topic possible. We hope that the insights reported in this Research Topic will inspire further research and promote new advances in the rapidly evolving field of Cellular Neuroscience.
